# Cell-like-carbon-micro-spheres for robust potassium anode

**DOI:** 10.1093/nsr/nwaa276

**Published:** 2020-11-07

**Authors:** Hongbo Ding, Jiang Zhou, Apparao M Rao, Bingan Lu

**Affiliations:** School of Physics and Electronics, State Key Laboratory of Advanced Design and Manufacturing for Vehicle Body, Hunan University, Changsha 410082, China; School of Materials Science and Engineering and Key Laboratory of Nonferrous Metal Materials Science and Engineering, Ministry of Education, Central South University, Changsha 410083, China; Department of Physics and Astronomy, Clemson Nanomaterials Institute, Clemson University, Clemson, SC 29634, USA; School of Physics and Electronics, State Key Laboratory of Advanced Design and Manufacturing for Vehicle Body, Hunan University, Changsha 410082, China; Fujian Strait Research Institute of Industrial Graphene Technologies, Quanzhou 362000, China

**Keywords:** bionic structures, potassium ion battery, reversible capacity, term stability, rate performance

## Abstract

Large-scale low-cost synthesis methods for potassium ion battery (PIB) anodes with long cycle life and high capacity have remained challenging. Here, inspired by the structure of a biological cell, biomimetic carbon cells (BCCs) were synthesized and used as PIB anodes. The protruding carbon nanotubes across the BCC wall mimicked the ion-transporting channels present in the cell membrane, and enhanced the rate performance of PIBs. In addition, the robust carbon shell of the BCC could protect its overall structure, and the open space inside the BCC could accommodate the volume changes caused by K^+^ insertion, which greatly improved the stability of PIBs. For the first time, a stable solid electrolyte interphase layer is formed on the surface of amorphous carbon. Collectively, the unique structural characteristics of the BCCs resulted in PIBs that showed a high reversible capacity (302 mAh g^−1^ at 100 mA g^−1^ and 248 mAh g^−1^ at 500 mA g^−1^), excellent cycle stability (reversible capacity of 226 mAh g^−1^ after 2100 cycles and a continuous running time of more than 15 months at a current density of 100 mA g^−1^), and an excellent rate performance (160 mAh g^−1^ at 1 A g^−1^). This study represents a new strategy for boosting battery performance, and could pave the way for the next generation of battery-powered applications.

## Teaser

A potassium ion battery with biomimetic carbon cells as the anode exhibits high reversible capacity, excellent cycle stability (continuous running time of over 15 months) and excellent rate performance.

## INTRODUCTION

Due to their inherently low cost and high energy density, potassium ion batteries (PIBs) are posited as the next-generation large-scale energy storage systems [1–7]. Recent in-depth exploration of advanced anode materials for PIBs [8–14] include the metallic materials [15–19], sulfides [20–24], selenides [25–28], polymers [29], phosphides [30–35] and carbon materials [36–42]. It is well known that the interaction of K^+^ with metallic electrodes results in a significant volume expansion; for example, Sb reacts with K^+^ to form K_3_Sb which suffers from a volume expansion of ∼300%. It is also well known in the battery literature that such volume expansion leads to the pulverization and fragmentation of the electrode material, which consequently results in a rapid decline in capacity and extremely poor cycle stability [43]. Additionally, other types of compound anodes have limited utility because of their short cycle life. The anodes used in commercial lithium ion batteries (LIBs) are mainly carbon-based materials [44–46], which hold promise as PIB anodes with long cycle life and high specific capacity [47–53]. In this regard we have already reported a few breakthroughs, see for example Ref [54]. However, their rate performance and rapid charge/discharge characteristics still fall short for practical battery applications, which warrants a continued search for advanced carbon-based anodes [5,45,51,55,56].

Over billions of years, biological cells evolved effectively by natural selection and resulted in the creation of a variety of organisms, and cells such as human cells that can be regarded as perfect small systems. The structure of such cells is complex yet delicate with various well-coordinated structural components; for example, the cell membrane provides access to biomaterials and can discharge metabolic waste in a timely manner [57,58]. Here we propose and demonstrate that such evolution-selected cells have important implications in the synthesis of battery materials. Previously, we demonstrated that metal ions selectively absorb and accumulate within the cells of halophytic plants, which then can be converted into graded three-dimensional carbon and metal oxide nanocomposites [59]. It is noteworthy that the plant cells can accommodate well their volume changes and mechanical stress. In subsequent work, we developed a micro-yolk shell structure of Mn_2_P_2_O_7_ by utilizing bacteria to absorb Mn^2+^, and then carbonized the bacteria to make superior electrode materials for the LIBs [60].

We hypothesize that encapsulated carbon nanomaterials with cell-like membranes that contain ion-transporting channels could enhance the structural stability, rate performance and long cycle life of PIBs. To this end, we synthesized biomimetic carbon cells (BCCs) akin to the biological cells with several ion-transporting channels. The carbon nanotubes serve as the ion channels in BCCs, which greatly enhance the diffusion rate of K ions. In addition to demonstrating the robustness of the carbon shell of a BCC, we also demonstrate that the presence of a small nitrogen dopant concentration in the nanotubes is beneficial for the insertion of K ions. Collectively, the unique structural characteristics of the BCCs enabled us to fabricate PIBs that showed a high reversible capacity (302 mAh g^−1^ at 100 mA g^−1^ and 248 mAh g^−1^ at 500 mA g^−1^), excellent cycle stability (reversible capacity of 226 mAh g^−1^ after 2100 cycles and a continuous running time of more than 15 months at a current density of 100 mA g^−1^), and an excellent rate performance (160 mAh g^−1^ at 1 A g^−1^). This study represents a new strategy for boosting battery performance, and could pave the way for the next generation of battery-powered applications.

## RESULTS AND DISCUSSION

**Figure 1.**
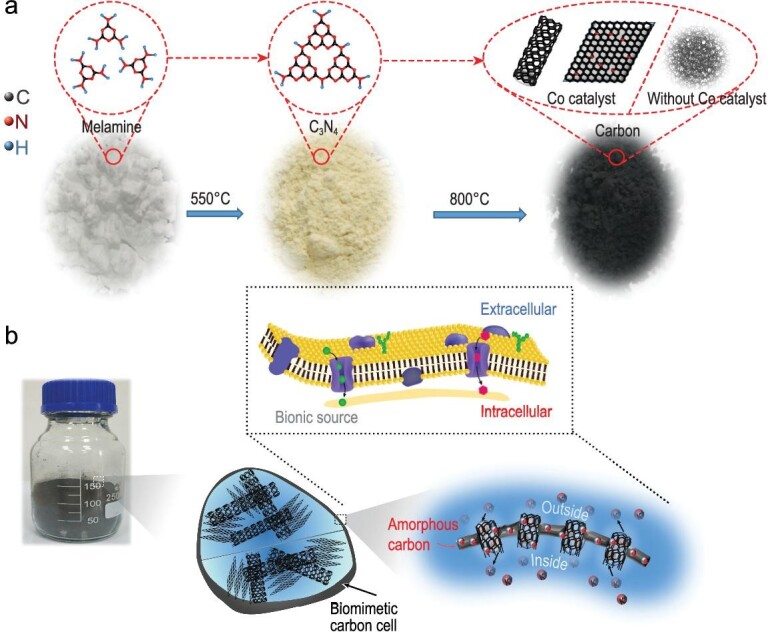
A schematic illustrating (a) the synthetic route for preparing BCCs, and (b) the structural and functional similarity of membranes in a biological cell and a BCC.

The synthesis of BCCs is schematically shown in Fig. 1a. The melamine precursor was heated in a muffle tube furnace to prepare a C_3_N_4_ intermediate (Supplementary Fig. S1a–d). Next, the intermediate and Co metal catalyst were placed inside the tube furnace and heated to 800°C in flowing Ar for 2 hours, and naturally cooled to room temperature. Finally, the product was etched in HCl to remove the metal catalysts to obtain the BCCs. Our synthesis method resulted in the formation of three types of catalytic carbon nanomaterials in the BCCs. First, the cobalt catalysts promoted the growth of carbon nanotubes whose diameter was determined by the size of the catalyst particles. At the same time, layers of graphene analogues were formed on the surface of the relatively larger cobalt particles (Supplementary Fig. S2a and b), and these products represent the graphite present in the BCC. Subsequently, amorphous carbon grew on the surfaces of the nanotubes and graphene, and protected the entire BCC. Interestingly, a detailed electron microscopic study revealed a striking similarity between the structure of a BCC and that of a biological cell. As shown in Fig. 1b, many organelles and internal spaces are present inside a biological cell, and its surface is composed of bilayer lipid membranes. The membranes contain many channels, which enable biological cells to exchange substances across its membrane. Likewise, the interior of a BCC contains open spaces and carbonaceous materials with carbon nanotubes (present on the surface and inside) to quickly transfer ions to any part of the BCC, thus greatly enhancing the electron transfer rate. Moreover, because of its internal space the BCC can accommodate the volume change caused by the K^+^ insertion into graphite, and the carbon shell of the cell-like membrane can keep the overall structure intact. These biomimetic characteristics of BCC are attractive for use as PIB electrodes.

**Figure 2.**
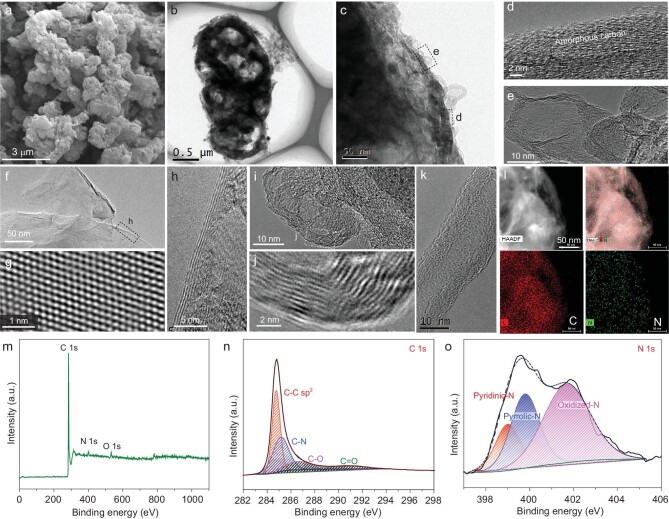
Morphological and structural characterization. (a) The scanning electron microscope (SEM) image of the BCCs. (b) The transmission electron microscope (TEM) image of a BCC. (c) Surface structure of a BCC. (d and e) Amorphous carbon and carbon nanotubes present on the surface of BCC. (f–h) Graphene and graphite-like materials present inside the BCC. (i–k) Carbon nanotubes present inside the BCC. (l) Elemental maps of the interior material present in a BCC which revealed the presence of C and N. (m) XPS survey spectrum, and the high-resolution XPS spectrum of (n) C 1s and (o) N 1s.

Figure 2 shows the morphology and structure of the BCCs. As shown in Fig. 2a and Supplementary Fig. S3a–c, the BCCs exhibit a characteristic ellipsoidal shape similar to that of the biological cells. A closer look at the BCCs reveals that their surface is composed of carbon shells akin to carbon nanotubes (Fig. 2b and Supplementary Fig. S3d–f), which can be readily discerned in Fig. 2c–e and Supplementary Fig. S3g and h, that also reveal the presence of graphitic and amorphous carbons on the surface of the BCCs. The protruding carbon nanotubes on the surface of the BCCs resemble the ion-transporting channels present in biological cells, and serve as a large number of K^+^ transporting channels (Supplementary Fig. S4a–d). In addition to facilitating fast ion transport, they also allow the electrolyte to infiltrate into the interior of the BCCs wherein few graphene-like flakes and crystalline graphite are present, which presumably contribute the most towards the measured specific capacity (Fig. 2f–l). Lastly, the energy-dispersive X-ray spectroscopy (EDS) revealed the presence of carbon and nitrogen in the interior material of the BCCs (Fig. 2l). The X-ray diffraction (XRD) pattern of the BCCs exhibited a sharp strong peak at 26.3^o^ (corresponding to the (002) planes of graphite) in addition to three minor peaks at 44.1^o^, 54.4^o^ and 77.5^o^, corresponding to the (101), (004) and (110) planes of graphite, respectively (Supplementary Fig. S5a). Consistent with the dominant XRD peak present at 26.3^o^, the Raman spectrum of BCC shows the characteristic *D* and *G* peaks at 1323 and 1581 cm^−1^, respectively (Supplementary Fig. S5b). The *D* peak is attributed to the presence of N dopants in the graphitic structure. A Brunauer–Emmet–Teller (BET) specific surface area of 40 m^2^ g^−1^ was determined from the nitrogen adsorption-desorption isotherms of the BCCs (Supplementary Fig. S5c). The protruding carbon nanotubes along with the porosity of BCC electrodes play a vital role in the electrochemical performance of PIBs, viz., the internal space within the BCC can accommodate a volume change caused by K^+^ insertion facilitated by the nanotubes, which greatly improves the K^+^ diffusion rate and the cycle stability of PIBs. The nature of bonding between the carbon, nitrogen and oxygen present in the BCCs was revealed through X-ray photoelectron spectroscopy (XPS), which exhibited significant C, N and O peaks at 285, 401 and 532 eV, respectively. The mass ratios of the three elements are 95.49%, 2.96% and 1.55%, respectively (Fig. 2m; Supplementary Table S1). In Fig. 2n, it is evident that the C 1s spectrum of the BCC can be fitted with four peaks that correspond to C–C (284.5 eV), C–N (285.2 eV), C–O (286.5 eV) and C=O (290.0 eV), respectively. Likewise, the three fitted peaks of the N 1s peak (Fig. 2o) correspond to the pyridinic-N (398.8 eV), pyrrolic-N (399.8 eV) and oxidized-N (401.7 eV). The pyridinic-N and pyrrolic-N exist at the electrochemically active sites as functional groups, which can increase the diffusion rate of K^+^. Oxidized-N exists inside the graphite plane, which greatly enhances the conductivity of the graphitic carbon. Collectively, the N dopants improve the capacity and rate performance of the BCCs.

**Figure 3.**
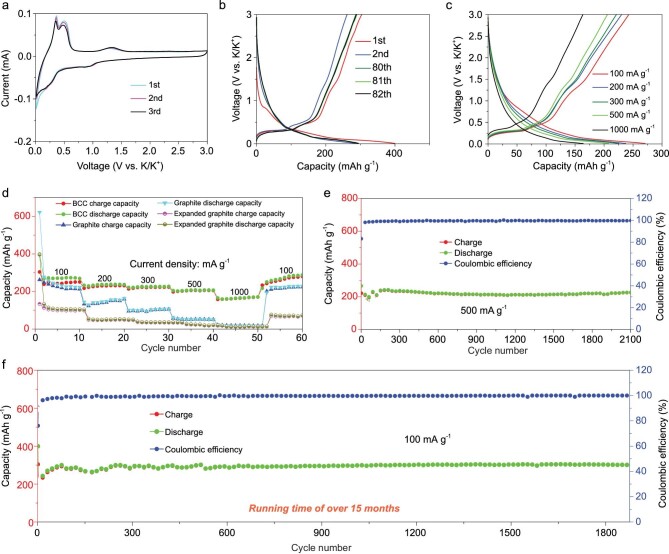
Electrochemical properties of the BCCs. (a) CV curves at a scan rate of 0.1 mV s^−1^ in the voltage range of 0.01–3.0 V. (b) Discharge and charge profiles at 100 mA g^−1^, and (c) at different current rates. (d) Rate performance of BCC, graphite and expanded graphite at various current densities. Long-term cycling performance at (e) 500 mA g^−1^ and (f) 100 mA g^−1^.

**Figure 4.**
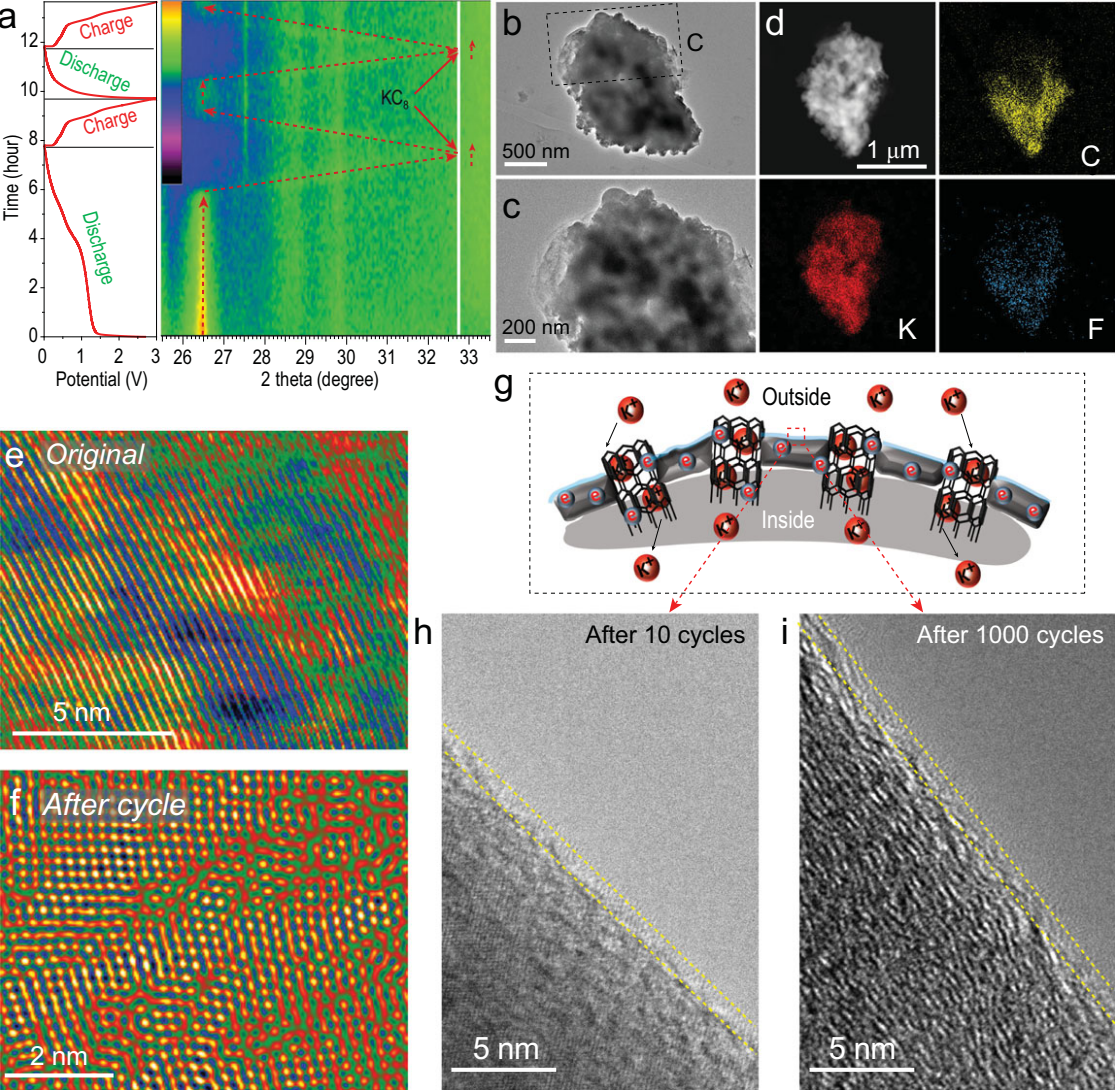
The structural evolution, morphological changes and elemental maps of the BCCs. (a) Galavanostatic charge–discharge curves of BCC anodes for the first two cycles, and the related *in situ* XRD patterns during cycling. (b) TEM image of a BCC after 1000 cycles. (c) Expanded view of the boxed region in (b). (d) EDS maps of the BCC electrode showing the distribution of C, K and F elements. (e and f) TEM images of graphite present in the BCC before and after 1000 cycles. (g) Schematic diagram and TEM image of the SEI layer on the BCC surface after (h) 10 cycles and (i) 1000 cycles.

The BCCs were used as PIB anodes, and their cyclic voltammetry (CV) and constant current charge and discharge characteristics revealed the K^+^ storage behavior in BCCs. Figure 3a shows the first three CV curves of a BCC electrode in the 0.01 to 3 V voltage window at a scan rate of 0.1 mV s^−1^. A strong scanning peak appeared at 0.03 V due to the insertion of K^+^ into the graphitic layer, which resulted in the formation of potassium intercalated graphite KC_8_ (discussed further in Fig. 4a below). In the subsequent anodic scanning, two scanning peaks appeared at 0.36 and 0.55 V due to the de-intercalation of K^+^ from the intercalated graphite. In the next two cycles, the scanning peaks of K^+^ intercalation/de-intercalation remained unchanged and consistent with the first cycles, which highlights the excellent cycle stability of the BCCs. The cathodic peaks at 0.25 and 0.9 V, and the anodic peak at 1.25 V are attributed to the interaction of K ions with N species and may reflect various binding energies [61]. Figure 3b shows the discharge and charge curves of the BCCs at a current density of 100 mA g^−1^, and the two plateaus that appear during charging are due to the de-intercalation of K^+^ from the graphite layer. This behavior is consistent with the CV response depicted in Fig. 3a, and the same can be said about the second charge/discharge curve. In addition, the charge/discharge curves of the 80th, 81th and 82th are also shown in Fig. 3b in which the degree of overlap of the charge and discharge curves is very high, thus confirming that the BCCs exhibit excellent cycle stability. The K^+^ insertion and extraction, and rate performance of the BCC were tested under different current densities. As shown in Fig. 3c, at different current densities, the potassiation and depotassiation curves of the BCC show similar voltage platforms. In addition, the rate performance of BCC confirmed reversible capacities of 250, 230, 223, 206 and 170 mAh g^−1^ at current densities of 100, 200, 300, 500 and 1000 mA g^−1^, respectively (Fig. 3d). Upon returning the current density to 100 mA g^−1^ (and after many cycles), the reversible capacity of the BCCs was restored to ∼287 mAh g^−1^. Interestingly, compared with graphite and expanded graphite, even at higher current density, it can still provide higher capacity, which further proves that BCC has excellent rate performance. Electrochemical impedance spectroscopy (EIS) is a technique widely used in the field of electrical analysis, which can prove the effectiveness of carbon nanotubes (CNTs) to enhance ion transmission. The semicircle of the curves is known to be related to the charge transfer resistance (Rct), which is mainly produced at the interface between the electrolyte and the electrode, which can explain the potassium ion transfer in the active electrode material particles. As shown in Supplementary Fig. S6, compared with the other two comparative graphite materials, BCCs have the lowest Rct, which means that the transfer resistance of potassium ions inside the BCC is the smallest. In addition, the rate performance of the battery can also illustrate the effectiveness of CNTs to enhance ion transmission from the side, maintaining high capacity even at a large current density. This evidence shows that CNTs play an important role in the process of ion transport. As such, the BCCs provide a large capacity even at a high current discharge, which is a key attribute for practical battery applications. Moreover, at a current density of 100 mA g^−1^ for continuous cycle testing, the BCCs exhibit excellent cycle stability (Supplementary Fig. S7a). It is also noteworthy that in most cycles, the battery's Coulombic efficiency reaches 99%, indicating that the BCCs exhibit superior rate performance. The long cycle performance of BCCs was tested at different current densities; for example, Fig. 3e and Supplementary Fig. S7b show a steady high capacity of 226 mAh g^−1^ when cycled at a higher current density of 500 mA g^−1^ for 2100 cycles. Compared to the initial reversible capacity, no loss in capacity was observed even after more than 2000 cycles, and in the first few cycles the Coulombic efficiency increased rapidly to 99%. Lastly, Fig. 3f and Fig. S7c show the long cycle stability of the BCC anode at a current density of 100 mA g^−1^ with a high capacity of 302 mAh g^−1^ and excellent cycle stability even after 1800 cycles. Importantly, this battery has been operating under these conditions for more than 15 months, which is on par with the cycle performance of commercial or experimental LIBs. In short, because of their unique structure the BCCs exhibit excellent cycle performance and stability at low or high current densities. In addition, in order to further verify the structural advantages of biomimetic carbon materials, BCC was used as the anode material of LIBs for cyclic testing. The test results show that BCCs can provide a high specific capacity when used as an LIB anode, and can also maintain excellent cycle stability (Supplementary Fig. S8). This also proves the advantages of the BCC structure from another aspect.

In order to further analyze the electrochemical behavior of the BCCs during charging and discharging, a kinetic study was conducted through CV curves at different scan rates. The CV curves of the BCC anode at 0.1, 0.3, 0.5 and 0.8 mV s^−1^ scan rates are dependent on the scan rates as seen in Supplementary Fig. S9a. With increasing scan rate, the redox peak intensity also increases and the anode potential gradually shifts in the positive direction while the cathode peak shifts in the negative direction. This behavior is due to the different storage methods of K^+^ ions at different scan rates. It is well known that the scan rate and the peak current are related [61] as

(1) \begin{equation*} i = a{v^b}, \end{equation*}

where *a* and *b* are constants, *i* is the peak current and ${v}$ is the scan rate. The *b* value can be obtained by plotting log(*i*) versus log(*v*) and computing the slope. The different values of *b* also have different meanings: a *b* value of 0.5 suggests that the electrochemical behavior of the electrode material is completely caused by diffusion, while a *b* value close to 1 indicates the presence of a surface capacitive behavior. As shown in Supplementary Fig. S9b, the *b* values calculated from the anodic peak are 0.61 and 0.89, and the *b* value of the cathodic peak is 0.68. This indicates that both the surface capacitive and diffusion processes are responsible for the storage of K^+^. Further, to quantitatively determine the capacitance contribution to the total electrode capacity, the peak current can be expressed as follows [61]:

(2) \begin{equation*} i = {k_1}v + {k_2}{v^{1/2}}, \end{equation*}

where *k*_1_ and ${k_{2}v^{1/2}}$ represent the capacitive and diffusion processes, respectively. Supplementary Fig. S9c shows that the capacitance contribution rate is 66.7% when the scan rate is 0.5 mV s^−1^, suggesting that the surface electrochemical behavior dominates at this scan rate. At 0.1, 0.3, 0.5 and 0.8 mV s^−1^, the capacitance contributions towards the BCC electrode capacity are 48.0%, 59.9%, 66.7% and 77.5%, respectively (Supplementary Fig. S9d).

The morphology, structure and elemental maps obtained after cycling BCCs as PIB anodes further confirmed the potassium storage mechanism described above, and the structural stability of BCCs during battery cycling (Fig. 4). The charge/discharge cycle at a current density of 100 mA g^−1^, and *in situ* XRD analysis of the BCC anode during charge and discharge was performed to investigate the structural changes of BCCs during the first two cycles. In order to explore the process of forming a stable solid electrolyte interphase (SEI) layer on the BCC surface, the surface of the negative electrode material tested *in situ* was not pre-potassized. As evident in Fig. 4a, the interlayer spacing of BCC changes during charging and discharging. During the first discharge the graphite peak present at 26.5^o^ disappears, and then reappears in the subsequent charge–discharge cycle indicating that (i) the K ions can be reversibly inserted and extracted from the BCC, and (ii) the structural stability of BCCs is excellent. In the *in situ* XRD spectrum, the 26.5^o^ diffraction peak upshifts continuously with the insertion of K ions. At the end of the discharge cycle, a new diffraction peak appears around 33^o^, which corresponds to the characteristic peak of KC_8_. In addition, the peaks at 27.5, 28.5 and 33^o^ correspond to different potassium-carbon compounds, including KC_48_, KC_24_, etc. [54]. The insertion of K^+^ into graphite will result in a volume change of the BCC, but the K^+^ can be reversibly extracted from the graphite as evidenced from the reappearance of the diffraction peak 26.5^o^, which underscores the excellent cycling stability of the BCCs. In addition, the BCCs were characterized by transmission electron microscope (TEM) after multiple cycles. As shown in Fig. 4b and c, and Supplementary Fig. S10a, the structure of a BCC remains unchanged even after 1000 cycles. Likewise, the lattice fringe images in Fig. 4e and Supplementary Fig. S10b reveal that the surface morphology of the BCC also remains robust, thus confirming the advantage of using BCCs in battery research and development. The high-angle annular dark-field (HAADF) images and corresponding EDS maps of the BCC anode when discharged to 0.01 V are shown in Fig. 4d, and provide strong evidence for that BCC stores a large amount of K^+^ ions. Furthermore, after cycling for 1000 cycles, the fully discharged BCC stores a large amount of K^+^ (Supplementary Fig. S10c and Table S2). Interestingly, F was also detected on the surface of the BCC due to the SEI layer formed on the surface. In addition, the microscopic morphology of graphite inside the BCC was also studied (Fig. 4e and f). One of the important reasons why BCCs can cycle continuously for a long duration is their structural stability, and consequently their stable SEI layer formed on the surface of the BCCs (Fig. 4g–i).

**Figure 5.**
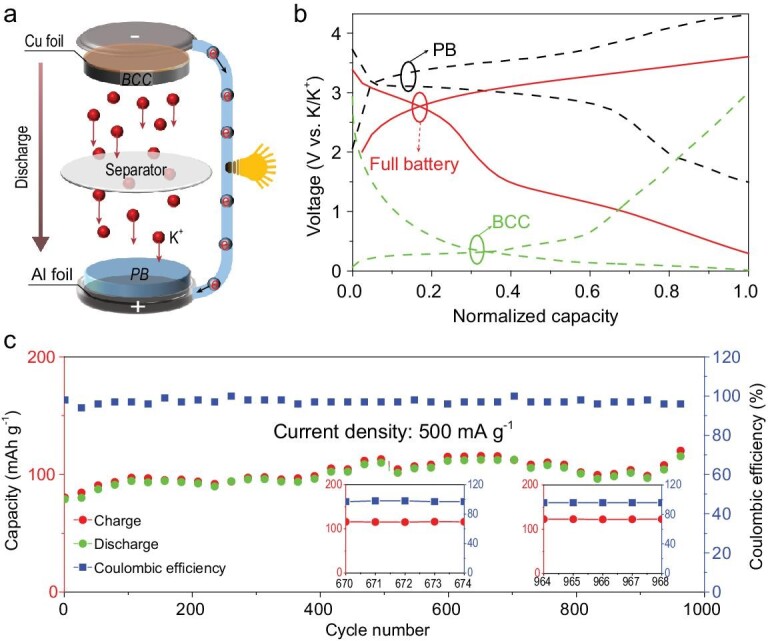
(a) Schematic illustration of the K-ion full battery based on the as-prepared BCC and Prussian Blue. (b) Charge–discharge profiles of the half battery and full battery. (c) Cycling stability at 500 mA g^−1^.

In order to further evaluate the prospects of BCC anodes for practical application, we assembled a full battery using BCC as the battery anode and Prussian Blue as the battery cathode. Figure 5a shows the working diagram of the full battery. The charge and discharge curves of the Prussian Blue cathode, BCC anode half-battery and the full battery assembled from the two materials are shown in Fig. 5b. It can be seen that the matched full battery has a suitable voltage range and charge/discharge voltage platform. More importantly, the assembled full battery has ultra-stable cycle performance, which is promising for commercial applications of PIBs. As shown in Fig. 5c, at a current density of 500 mA g^−1^, the K-ion full battery can provide an initial discharge capacity of 80 mAh g^−1^. After a short cycle of activation, the full battery capacity can reach 115 mAh g^−1^ (based on the anode mass). After about 1000 cycles, it can maintain an ultra-high capacity retention rate (compared to the highest capacity during the cycle), and the Coulombic efficiency can be as high as 98% during the stable cycle of the battery (the inset of Fig. 5c). Collectively, this study consistently found BCCs to exhibit excellent electrochemical performance when used as anodes in a half-battery or a full-battery. Thus, BCCs, with their ideal morphology and robust structure, could point the way for further development of high performance PIBs.

## CONCLUSION

In conclusion, we synthesized carbon materials similar to biological cells in a simple and cost effective way. BCCs are composed of carbon sheets with a high degree of graphitization and CNTs. CNTs connect the inside and outside of carbon cells, providing a large number of ion channels. A large number of ion channels increase the diffusion paths of ions and increase the transmission rate. The internal space possessed by the BCC provides a buffer for the volume change caused by the insertion of potassium ions into the graphite. The Carbon shell of the cell-like membrane can protect and support the internal materials and the overall structure, which greatly improves the cyclic stability of PIBs. The BCC-based electrodes demonstrated a superior cycling stability with a reversible capacity of 226 mAh g^−1^ after 2100 cycles at a current density of 500 mA g^−1^ and continuous running time of more than 15 months at a current density of 100 mA g^−1^. This work provides a new way for the design and manufacture of new biomimetic battery materials in the future, and promotes collaborative research across multiple disciplines.

## Supplementary Material

nwaa276_Supplemental_File
