# Supplementary material for: Cell-like-carbon-micro-spheres for robust potassium anode
Source: Natl Sci Rev. 2020 Nov 7;8(9):nwaa276. doi: 10.1093/nsr/nwaa276 (PMC8433086; doi:10.1093/nsr/nwaa276)
Supplement: nwaa276_Supplemental_File [file nwaa276_supplemental_file.docx]

**Supplementary Data**

**Cell-like-carbon-micro-spheres for Robust Potassium Anode**

*Hongbo Ding,^a^* *Jiang Zhou,^b,^** *Apparao M. Rao^c^ and* *Bingan Lu,^a,d,^**

*^a^* School of Physics and Electronics, State Key Laboratory of Advanced Design and Manufacturing for Vehicle Body, Hunan University, Changsha 410082, P. R. China

*^b^* School of Materials Science and Engineering and Key Laboratory of Nonferrous Metal Materials Science and Engineering, Ministry of Education, Central South University, Changsha 410083, PR China

*^c^* Department of Physics and Astronomy, Clemson Nanomaterials Institute, Clemson University, Clemson, SC, 29634 USA

*^d^* Fujian Strait Research Institute of Industrial Graphene Technologies, Quanzhou 362000, P.R. China

* Email: [zhou_jiang@csu.edu.cn](mailto:zhou_jiang@csu.edu.cn) and [luba2012@hnu.edu.cn](mailto:luba2012@hnu.edu.cn)

**Experimental Section**

**Preparation of BCC**

Synthesis of carbonitrides: In this study, ~15 mg of melamine powder was placed in a porcelain boat and heated to 550 °C in a muffle tube furnace with a heating rate of 2 °C min^–1^ in air. The powder was held at 550 °C for 5 h and allowed to cool naturally to room temperature. A yellow colored product was retrieved from the porcelain boat, ground into a fine powder in an agate mortar (referred below as the carbon-nitrogen compound) and used as the precursor for preparing BCC.

Synthesis of BCC: The yellow colored carbon-nitrogen powder was mixed with commercial Co catalyst (mass ratio of 1: 1), and thoroughly ground in an agate mortar. Next, the mixed powder was transferred to a porcelain boat and heated to 800 °C in Ar atmosphere with a heating rate of 2 °C min^–1^. After 2 h, the furnace was allowed to cool naturally to room temperature. The final product was washed with 2 mol L^–1^ HCl to remove the Co catalyst, and then rinsed thrice with deionized water. After centrifuging the washed product, it was placed in a 60 °C vacuum drying oven and dried overnight to obtain the BCC powder.

**Material Characterization**

The morphology of the BCC was investigated by transmission electron microscopy (TEM, Titan G2 60-300 with image corrector). Field emission scanning electron microscope (FESEM, Hitachi S-4800, 20 kV) images revealed the shape of the BCC, and the elemental content of BCC was revealed through energy dispersive spectroscopy (EDS). The crystalline phase of the BCC was analyzed by powder X-ray diffraction (XRD, Rint-2000, Rigaku, Cu Kα) and the ratio of disordered to graphitic carbon was determined through Raman spectroscopy (Renishaw 2000 system). The *in situ* XRD data (discussed in Fig. 4a for a battery operating with a current density of 100 mA g^–1^) was obtained using a Bruker D8 ADVANCE (Cu Kα) diffractometer.

**Electrochemical measurements**

The electrochemical performance of BCCs was evaluated by preparing coin cells (CR2032). As a first step, the active BCC material, conducting agent (acetylene black) and PVDF binder were mixed (weight ratio of 8:1:1) in N-methyl-2-pyrolidone (NMP) (AR, 99 %, Aladdin) solution and stirred overnight. Next, the anodes were prepared by spreading the slurry onto a copper foil current collector, which was then dried at 80 °C for 24 h. The active material loading was about ~ 1.5 mg cm^–2^. The cathodes were prepared in a similar fashion on Al foils with the prussian blue, conducting agent (acetylene black) and PVDF binder mixed in the weight ratio of 6:3:1. The material loading was about ~ 1 mg cm^–2^. The glass fiber (Whatman, GF/D) filters were used as separators and the 2032-type coin cells were assembled and disassembled in an argon-filled glove box (moisture and oxygen concentrations maintained below 0.5 ppm), pure potassium foil was used as the reference/counter electrode. The potassium bis(fluorosulfonyl)amide (KFSI, purity 98 %, 4 mol L^–1^) in an 1,2-Dimethoxyethane (DME) was used as the electrolyte. The assembly steps of a full battery are slightly different from those of a half battery. First, the anode material BCC and potassium metal are assembled according to the half-cell assembly method. The assembled half-cell is charged and discharged for two cycles on the battery test system, and the final state of the battery is in a fully discharged state. After the cycle is completed, the battery is disassembled in the glove box to take out the electrode, and then matched with the Prussian blue electrode to assemble a full battery. The battery assembly steps used in the in-situ XRD test are similar to half-cells. The battery mold used in the in-situ test is a special test equipment. Place the anode material electrode on the bottom of the mold, place the glass fiber separator on the material, drop a certain amount of electrolyte, and place the cut potassium metal sheet. After the assembly is completed, it is placed on the in-situ XRD test system for charging and discharging test and in-situ test at the same time. The rate performance and galvanostatic charge/discharge of the PIBs were performed using the Arbin Instrument BT 2000 model (Neware BTSCT-3008-TC 5.X. Shenzhen. China). Cyclic voltammetry (CV) was performed on the Chenhua CHI660E electrochemical workstation. All half-battery performance tests were carried out after pre-potassiating the electrodes as follows: place the potassium foil on the electrode, add few drops of the electrolyte and let it stand for about 1 h before assembling the battery.


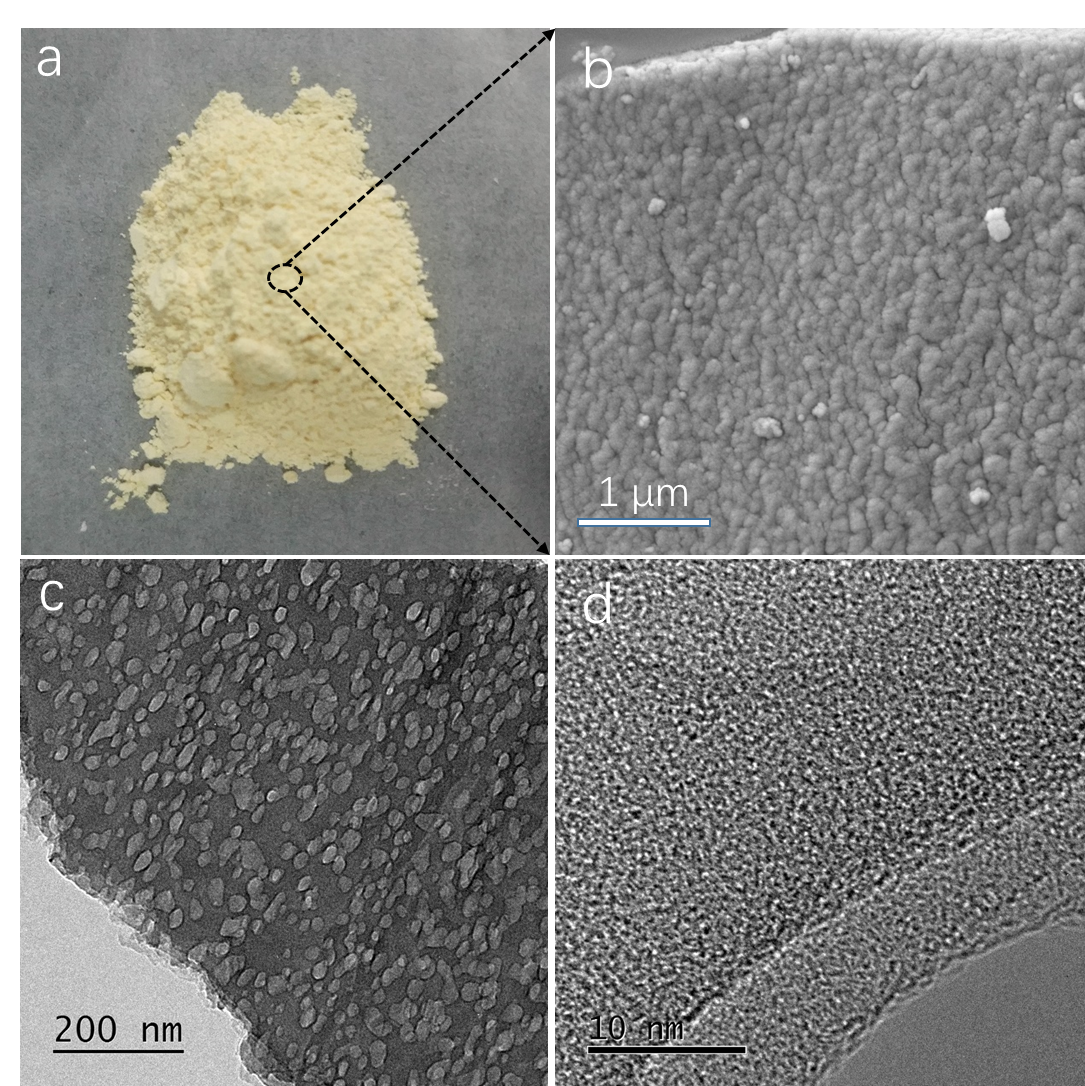


**Figure S1.** **(a) Picture of real products. (b) SEM, c) TEM, and d) HRTEM images of the C3N4 precursor.**


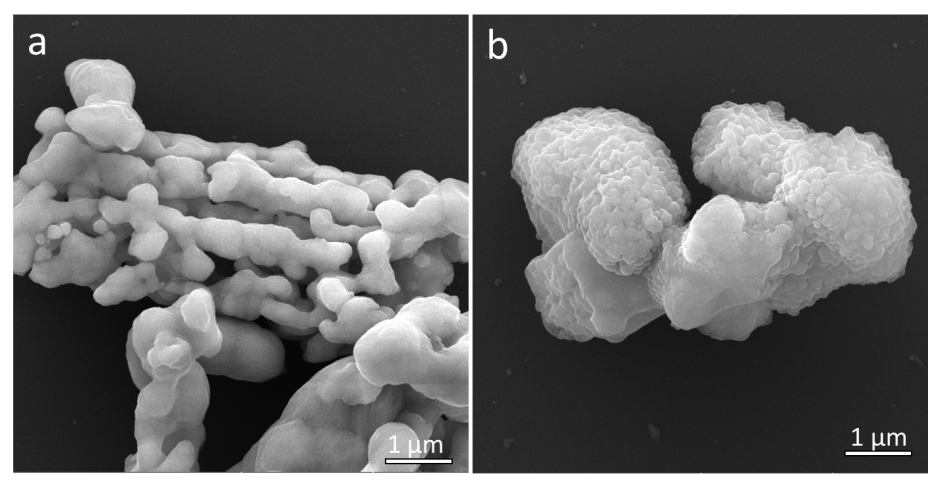


**Figure S2.** **(a, b) SEM images of cobalt catalyst particles.**

**
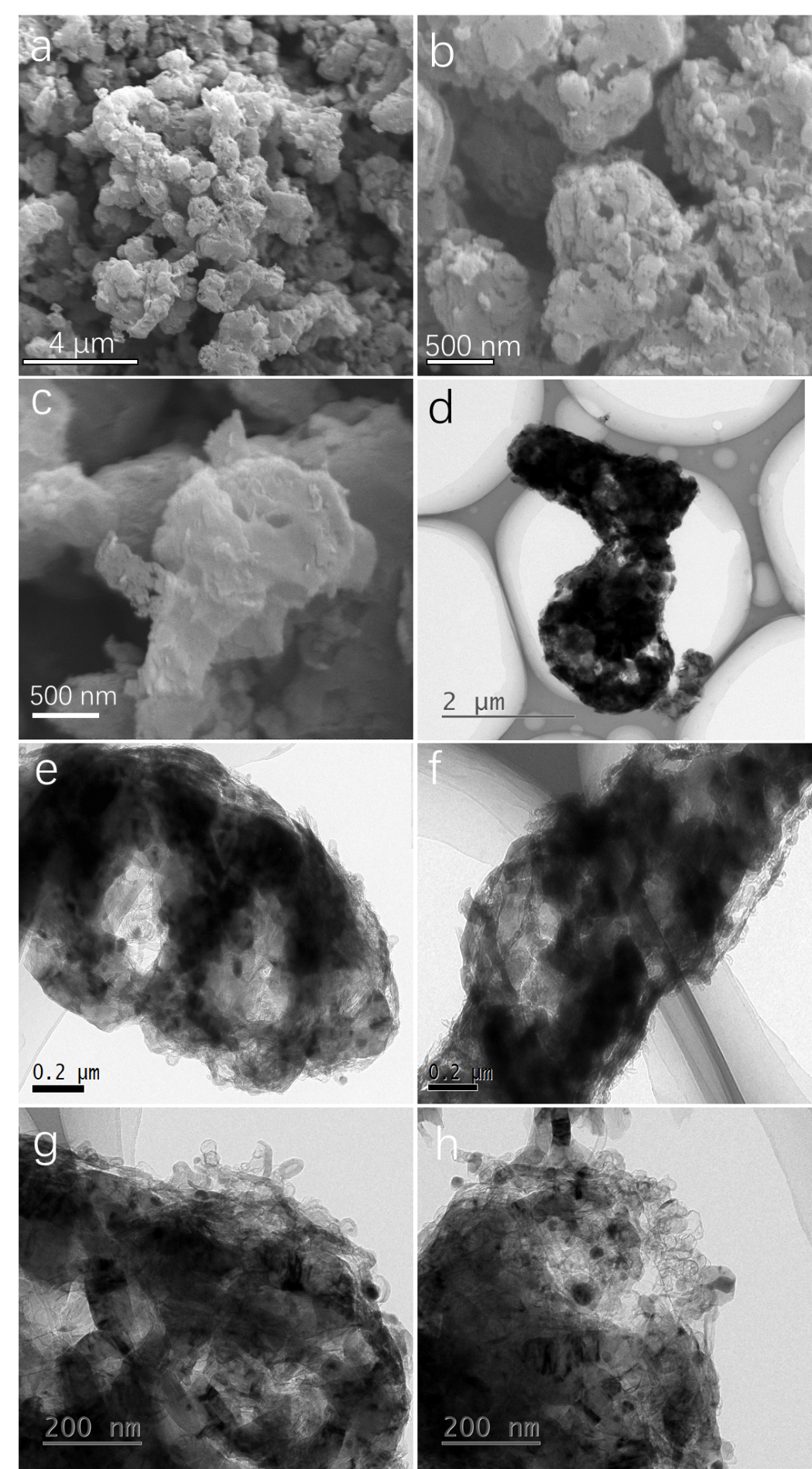
**

**Figure S3.** **(a, b, c) SEM images and** **(d, e, f) TEM images of the BCCs. (g, h) HRTEM images of the BCC surface.**


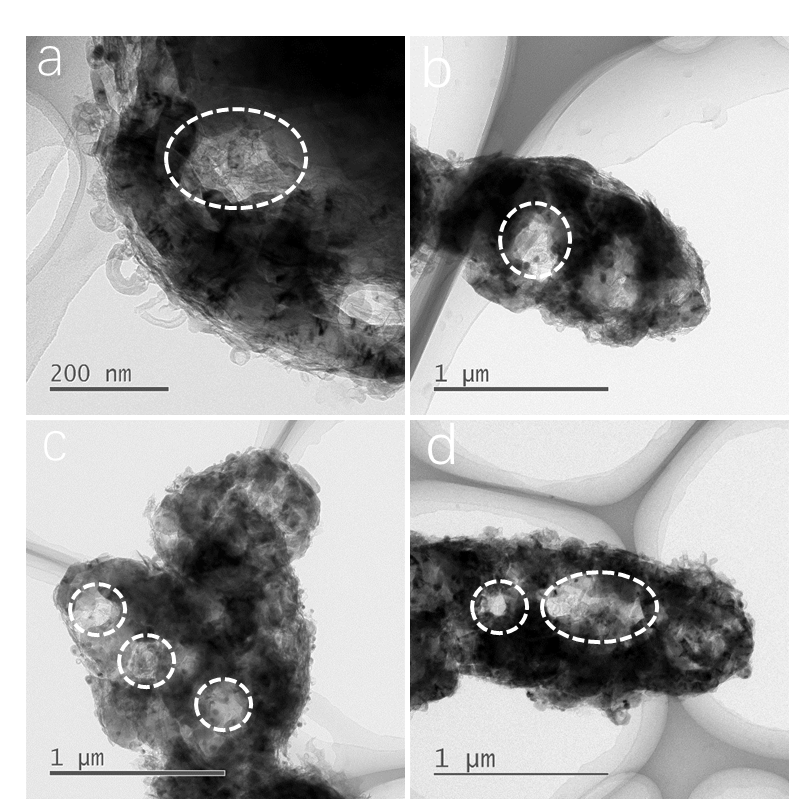


**Figure S4.** **(a, b, c, d) TEM images of the BCCs.**

**
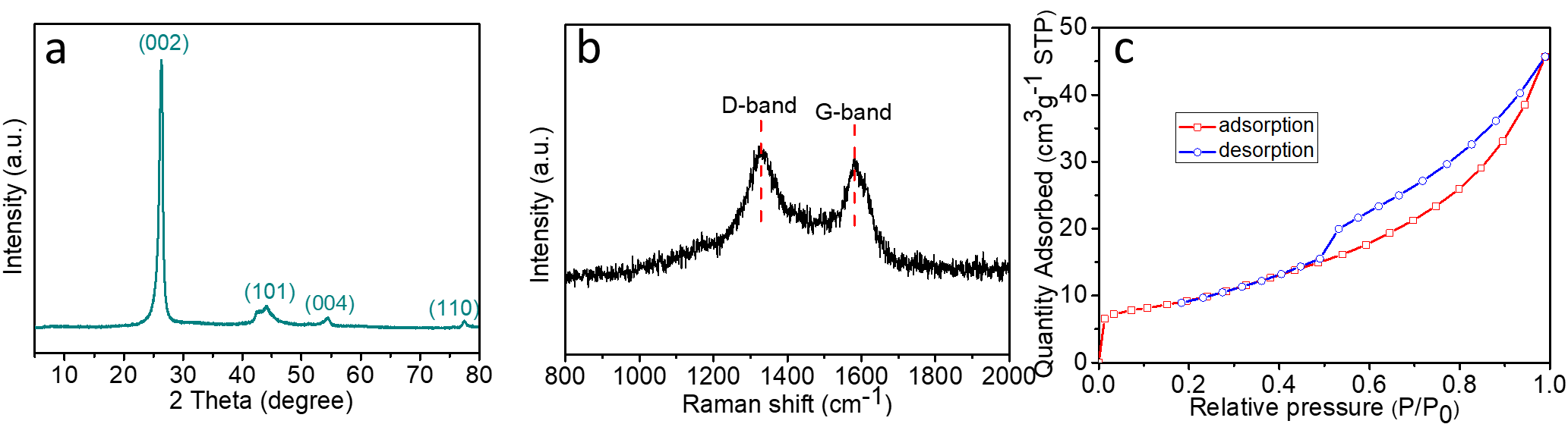
**

**Figure S5.** **Structural characterization of the BCCs.** **(a) XRD pattern. (b) Raman spectrum. (c) Nitrogen adsorption-desorption isotherms.**

| **Name** | **Peak BE** | **FWHM eV** | **Area(P) CPS.eV** | **Atomic %** |
| --- | --- | --- | --- | --- |
| **C1s** | **284.80** | **1.01** | **59987.50** | **95.49** |
| **O1s** | **532.55** | **3.34** | **2568.56** | **1.55** |
| **N1s** | **399.89** | **2.64** | **3008.79** | **2.96** |

**Table S1.** **Elemental composition of BCC as deduced from XPS.**


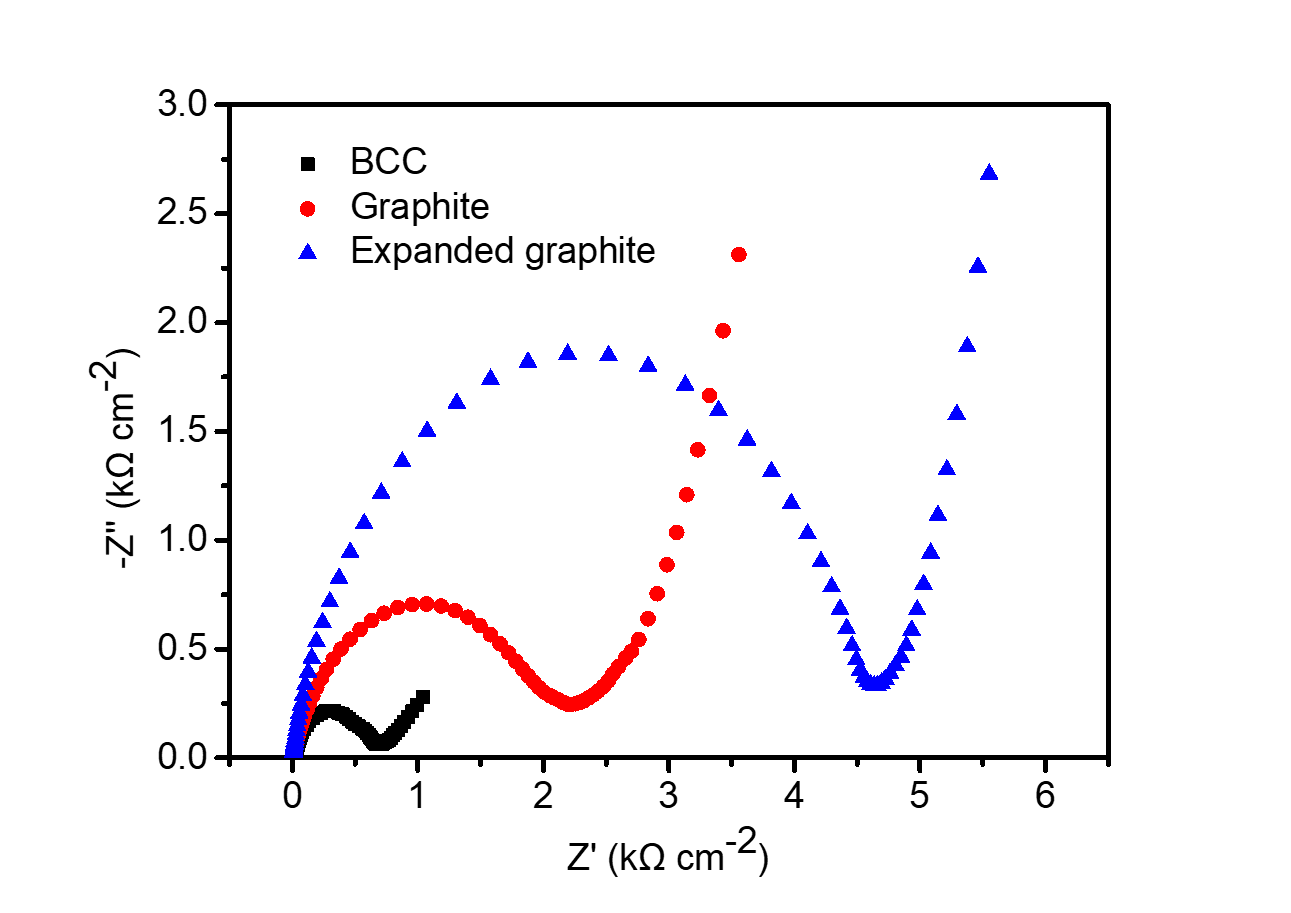


**Figure S6. EIS of the different carbon materials.**


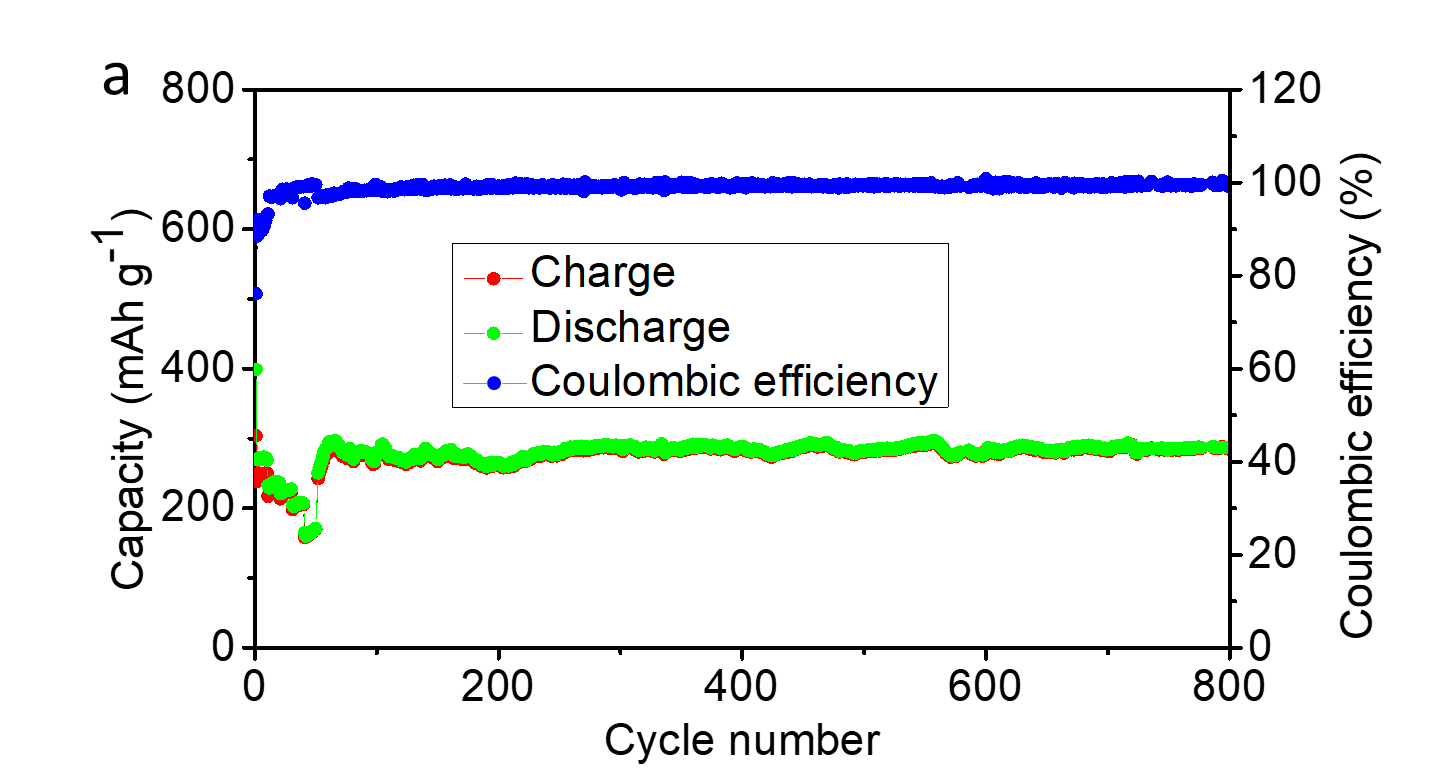


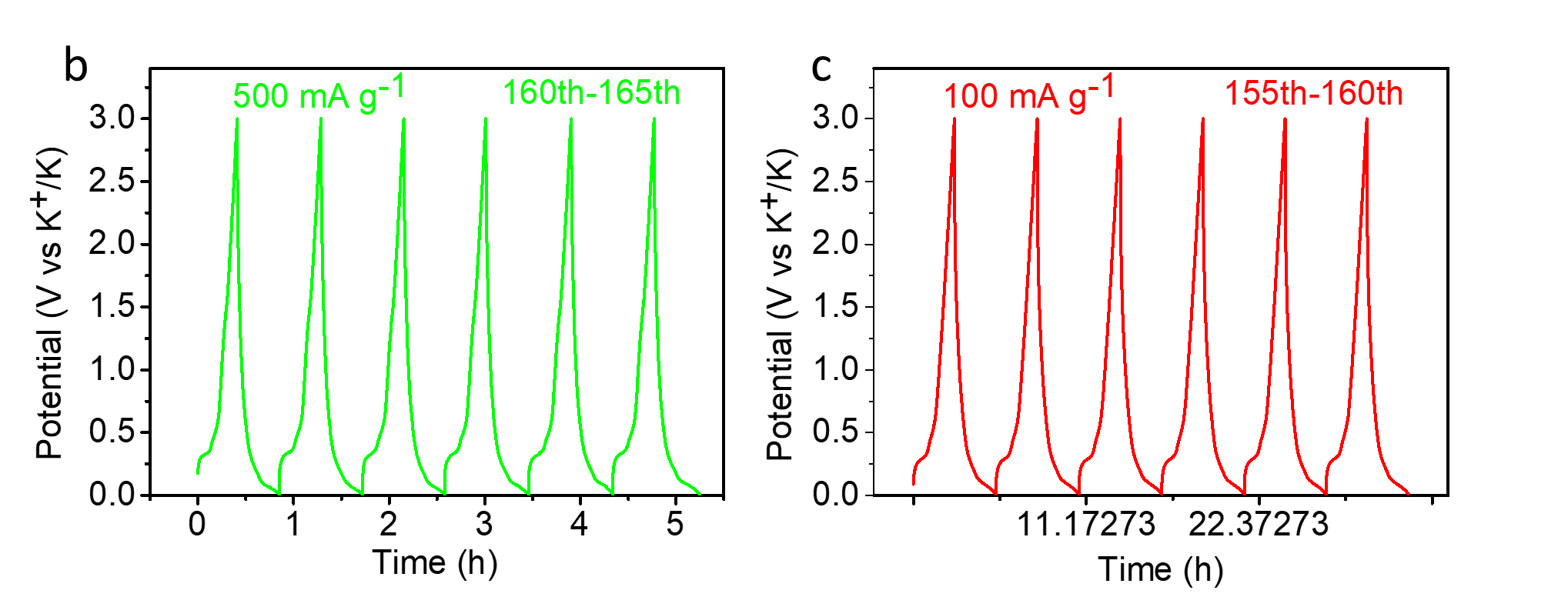


**Figure S7. (a) The long cycle performance of BCCs at a current density of 100 mA g^–1^. (b, c) Time-potential curves at different current densities.**

**Figure S8.** **Cycle performance of using BCC as anode of lithium ion battery.**


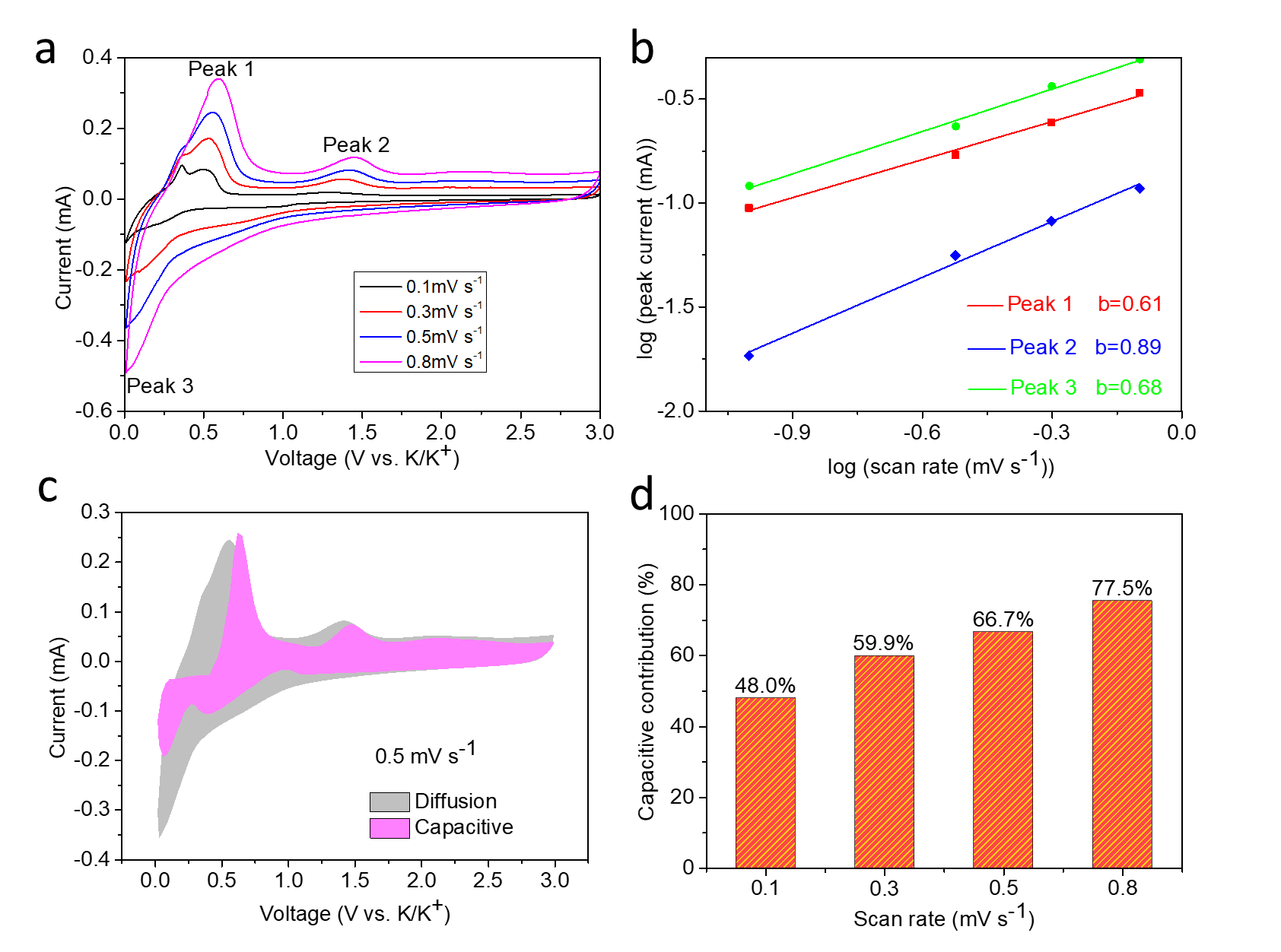


**Figure S9. (a) The cyclic voltammograms of BCCs at various scan rates. (b) *b* values of anodic and cathodic peaks. (c) CV curve collected at a scan rate of 1.0 mV s^–1^ with the pseudocapacitive fraction shown by the purple region. (d) The contribution of the capacitance in the BCC at different scan rates.**


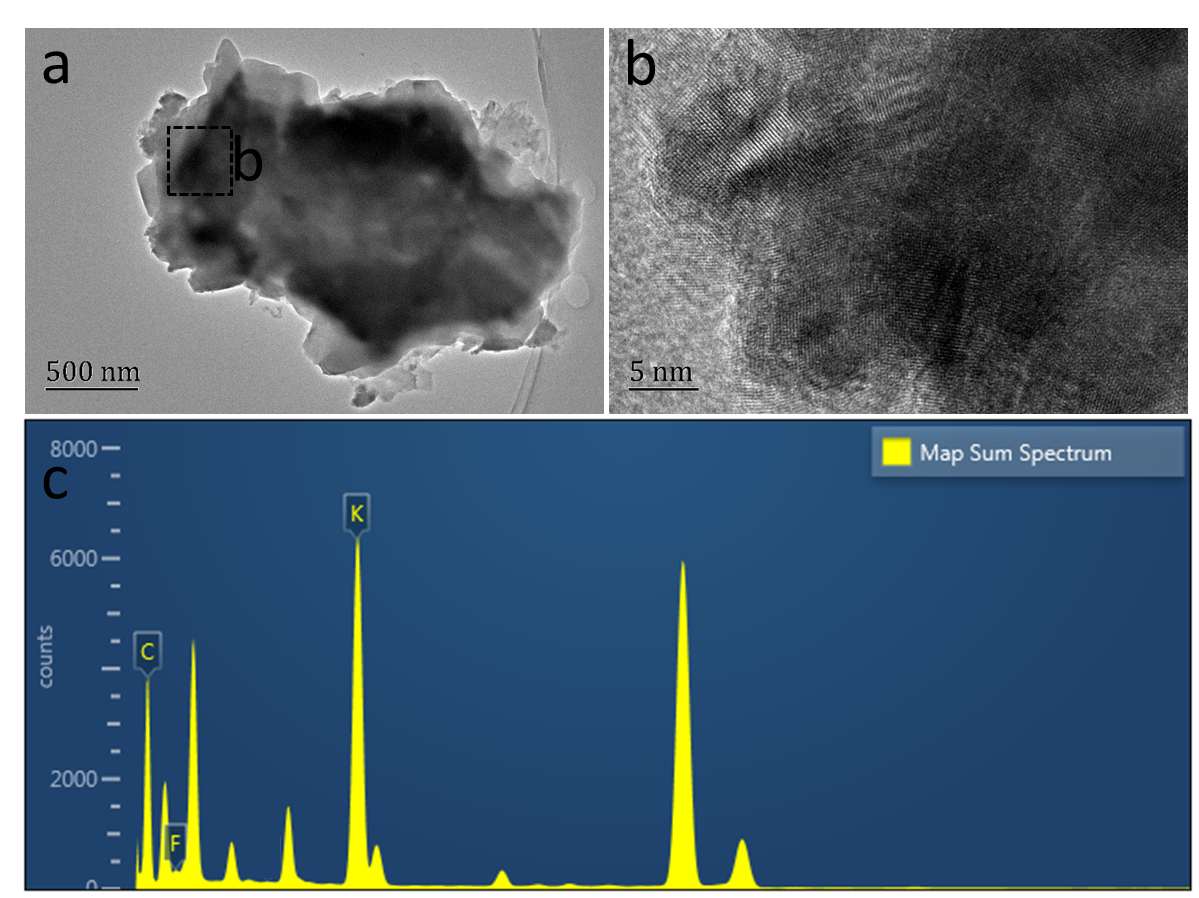


**Figure S10.** **(a, b) TEM image of a BCC** **after 1000 cycles. (c) Elemental composition of BCC** **after 1000 cycles.**

| **Element** | **Line Type** | **k Factor** | **Absorption Correction** | **Wt%** | **Wt% Sigma** |
| --- | --- | --- | --- | --- | --- |
| **C** | **K series** | **2.769** | **1.00** | **45.62** | **0.51** |
| **K** | **K series** | **1.009** | **1.00** | **51.74** | **0.49** |

**Table. S2.** **Elemental ratio in the BCC after 1000 cycles.**
